# Communication Among Photoreceptors and the Central Clock Affects Sleep Profile

**DOI:** 10.3389/fphys.2020.00993

**Published:** 2020-08-11

**Authors:** Milena Damulewicz, Juan I. Ispizua, Maria F. Ceriani, Elzbieta M. Pyza

**Affiliations:** ^1^Department of Cell Biology and Imaging, Jagiellonian University, Kraków, Poland; ^2^Laboratorio de Genética del Comportamiento, Fundación Instituto Leloir, IIBBA-CONICET, Buenos Aires, Argentina

**Keywords:** sleep, photoreceptors, Hofbauer-Buchner eyelets, *Drosophila*, peripheral clock

## Abstract

Light is one of the most important factors regulating rhythmical behavior of *Drosophila melanogaster*. It is received by different photoreceptors and entrains the circadian clock, which controls sleep. The retina is known to be essential for light perception, as it is composed of specialized light-sensitive cells which transmit signal to deeper parts of the brain. In this study we examined the role of specific photoreceptor types and peripheral oscillators located in these cells in the regulation of sleep pattern. We showed that sleep is controlled by the visual system in a very complex way. Photoreceptors expressing Rh1, Rh3 are involved in night-time sleep regulation, while cells expressing Rh5 and Rh6 affect sleep both during the day and night. Moreover, Hofbauer-Buchner (HB) eyelets which can directly contact with s-LN_*v*_s and l-LN_*v*_s play a wake-promoting function during the day. In addition, we showed that L2 interneurons, which receive signal from R1-6, form direct synaptic contacts with l-LN_*v*_s, which provides new light input to the clock network.

## Introduction

Circadian rhythms in *Drosophila* are regulated by a system of oscillators, which includes the pacemaker located in the central brain and peripheral oscillators located in various cells, tissues, and organs. Peripheral oscillators such as the ones in glial cells, compound eyes, antennae, gustatory receptor neurons, or Malpighian tubules express clock genes and show circadian rhythms in their structure and physiological processes (Siwicki et al., 1988; Zerr et al., 1990; Hege et al., 1997; Krishnan et al., 1999; Chatterjee and Hardin, 2010; Chatterjee et al., 2010).

The compound eye consists of ommatidia and each of them contains eight photoreceptors. Six of them, R1–R6, are located in the distal retina and express rhodopsin 1 (Rh1), sensitive to a broad spectrum of light wavelengths (O’Tousa et al., 1985; Hardie, 1987). R1–R6 terminate in the lamina, where they form tetrad synaptic contacts with L1, L2, L3, and amacrine cells (Meinertzhagen and O’Neil, 1991; Meinertzhagen and Sorra, 2001). The R1–R6 photoreceptors are involved in motion detection and image formation (Rister et al., 2007; Yamaguchi et al., 2008). Two other photoreceptors of each ommatidium, R7 and R8, are involved in color vision and detection of polarized light. They terminate in the second optic neuropil (medulla), where they contact transmedulla neurons (Tm5, Tm9, and Tm20; Gao et al., 2008; Melnattur et al., 2014). Tm5, Tm20 neurons receive also indirect input from R1–R6, through L3 (Gao et al., 2008; Melnattur et al., 2014). R7 forms additional synaptic contacts with amacrine cell Dm8 (Gao et al., 2008), and moreover, R7 and R8 contact each other in the medulla through direct synaptic contacts (Takemura et al., 2015), most probably using histamine as a neurotransmitter (Hardie and Raghu, 2001; Pantazis et al., 2008; Schnaitmann et al., 2018). R7 photoreceptors express UV-sensitive rhodopsin 3 (Rh3) or blue light – absorbing Rh4, while R8 cells express Rh3, blue light-sensitive Rh5 or green light-absorbing Rh6 (Salcedo et al., 1999).

According to the rhodopsin type expressed in R7 and R8, three different subpopulations of photoreceptors have been described: “pale” ommatidia consist of Rh3-expressing R7 and Rh5-expressing R8, the “yellow” type is composed of Rh4 expressing R7 and Rh6-expressing R8, and “DRA” (dorsal rim area) expresses Rh3 in both R7 and R8 and is involved in the polarized light detection (Wernet et al., 2006). An additional photoreceptive structure in the visual system is the Hofbauer–Buchner (HB) eyelets (Hofbauer and Buchner, 1989; Yasuyama and Meinertzhagen, 1999) composed of 4 cells expressing Rh6, terminating in the accessory medulla (Helfrich-Förster et al., 2002).

Different photoreceptor types and photopigments seem to play different roles in the circadian rhythm and behavior regulation. R1–R6, expressing Rh1, play a role in dim light detection in motion vision and phototaxis; they are also important for nocturnal activity which increases in response to raising day-light activity (Schlichting et al., 2014). Rhodopsin 6, expressed in a population of R8 photoreceptors, plays a role in the integration of light signals received by the other photoreceptors (Saint-Charles et al., 2016), while Rh5-expressing cells seems to be involved in light entrainment, that is the clock ability to adapt to light cycles and phase shifts of the rhythm, which suggests that Rh5 may use NorpA-independent pathway (Saint-Charles et al., 2016). Finally, HB eyelets play a role in regulating evening onset under high intensity light conditions, as well as the length of the siesta (Schlichting et al., 2019).

The role of the visual system in circadian entrainment has already been studied (Nippe et al., 2017), however, the effect of different photoreceptors on sleep is still not fully recognized. In this study we examined the role of different photoreceptors and their postsynaptic targets in the regulation of sleep and locomotor activity. We have shown that Rh1 and Rh3-expressing photoreceptors affect sleep during the night, while Rh5 and Rh6-expressing cells, both during the day and night. Moreover, we have presented that photoreceptors influence the pace of the molecular clock in pacemaker cells and that retinal oscillators are as much important for maintaining sleep as synaptic transmission from photoreceptors to target cells. Finally, we have described connections between the visual system and clock cells.

## Materials and Methods

### Fly Strains

The following strains of *Drosophila melanogaster* were used in the present study: GMR-Gal4, *Rh1*-Gal4, *Rh3*-Gal4, *sp/Cy0; Rh5*-Gal4, *yw; Sp/Cy0; Rh6*-Gal4/TM6B, R82F12AD/Cy0; R75H08DBD/TM6B (herein called *L2*-Gal4), *yw; Pdf*-Gal4, *w*; UAS-Δ*cyc24, w*; UAS-*TeTxLC* (herein called UAS-*TeTx*), *w; Pdf-*LexA,AS*-GFP_1–10_/Cy0;* LexAop*-GFP_11_* (for GRASP experiment), *yw*,UAS*-myrGFP*,QUAS*-HA::RFP; transTANGO* (herein called *transTANGO*), *w*; UAS-*GCaMP6f*, w; *Pdf*-LexA,LexAopGFP_11_; UAS-Nrx::GFP_1–10_ (for Nrx GRASP experiment).

L2-Gal4 strain was obtained from Janelia Research Campus, the others from Bloomington Drosophila Stock Center.

Flies were maintained under 12 h of light and 12 h of darkness (LD12:12) conditions and at a constant temperature of 25°C, unless the procedure required constant darkness (DD).

### Recording Locomotor Activity and Sleep

The locomotor activity was recorded using a monitoring system (TriKinetics) composed of monitors equipped with infrared light-emitting diodes and detectors, connected to a computer. Each monitor houses 32 glass-tubes of a diameter just sufficient to maintain a single fly. Tubes are sealed at both ends: one by food and the other by a foam stopper. When the fly passes the emitter/detector pair, the infrared beam is interrupted resulting in a signal transmitted to the computer. To analyze circadian rhythms in locomotor activity, flies were maintained for 7 days under LD12:12 and next under constant darkness (DD) for next 7 days.

Activity was counted every 5 min (1 bin) and analyzed in Excel by using “Befly!” software (Department of Genetics, University of Leicester). Lomb–Scargle normalized periodogram was used to determine rhythmic flies; flies with period value lower than 10 (confidence level 0.05) were regarded as arrhythmic. Flies which did not survive until the end of experiments were removed from analyses. Every experiment was repeated three times, at least 60 flies in total were used.

The Anticipation Index (Morning and Evening) was calculated for individual flies under LD12:12, by determining the proportion of activity counts during the 3 h preceding the phase transition over the activity within 6 h preceding phase transition.

To study sleep pattern, activity of flies was analyzed in the second day in LD12:12. Sleep was measured as intervals of at least 5 min of inactivity.

Heterozygous parental strains were used as control. In case of gene silencing, progeny of driver line crossed with UAS-VALIUM10-GFP was used as additional control. This strain has expression of empty VALIUM10-GFP vector in targeted cells.

Statistical analysis was performed using ANOVA with a Tukey’s multiple comparison test for normally distributed data. To analyze rhythmicity of flies we used non-parametric Kruskal–Wallis test to compare percentage from three repetitions. GraphPad Software was used to performed statistical analysis.

### Immunohistochemistry

Flies were decapitated and their heads were fixed in 4% paraformaldehyde in phosphate buffer saline (PBS; pH 7.4) for 4 h, then they were washed in PBS twice and cryoprotected by incubation in 12.5% sucrose for 10 min and in 25% sucrose at 4°C overnight. Material was embedded in Tissue Tek, frozen in liquid nitrogen, and cryostat 20 μm sections were cut. The sections were washed in PBS for 30 min and five times in phosphate buffer with an addition of 0.2% Triton X100 (PBT). After that, sections were incubated in 5% normal goat serum (NGS) with an addition of 0.5% bovine serum albumin (BSA) for 30 min at room temperature. Next they were incubated with primary antibodies for 24 h. Afterwards, sections were washed six times in PBT/BSA, blocked in 5% NGS for 45 min and secondary antibodies were applied for overnight incubation at 4°C. Finally, sections were washed twice in BSA, six times in PBT, and twice in PBS. Then, cryosections were mounted in Vectashield medium (Vector) and examined with a Zeiss Meta 510 Laser Scanning Microscope.

In addition to sections of the brain, whole brains were also used for immunohistochemistry. They were isolated after 1 h of head fixation in 4% PFA, washed in PBS and fixed again for the next 45 min. The next steps of immunostaining were done according to the protocol described above for cryosections.

For immunohistochemistry the following antibodies were used: nc82 (against the presynaptic protein Bruchpilot) (1:20, Developmental Studies Hybridoma Bank), PDF C7 (against Pigment Dispersing Factor) (1:500, Developmental Studies Hybridoma Bank), anti-GFP (rabbit, 1:1000, Novus Biological), anti-GFP (mouse, 1:20, Sigma Aldrich), goat anti-mouse conjugated with Cy3 (1:500, Jackson ImmunoResearch Laboratories, Inc.), goat anti-rabbit conjugated with Alexa 488 (1:1000, Molecular Probes), goat anti-mouse conjugated with Cy2 (1:500, Abcam).

### Calcium Imaging

L2>*GCaMP6f* flies were dissected on ice at specific time points, and brains were placed in PBS. Images of the brain were collected immediately with a confocal microscope. The fluorescence intensity was measured using ImageJ software. The ratio of fluorescence per area was calculated using ImageJ macro. Data obtained at different time points were compared.

### TransTANGO

L2-Gal4 or GMR-Gal4 crossed with *transTANGO* flies were raised at 18°C, adult males were separated and aged for 15 days at 18°C. The ICCs procedure was the same as described above, except for the length of the incubation with primary antibody, which was extended to 5 days at 4°C. The following primary antibodies were used: rabbit anti-DsRed (1:250, Rockland), chicken anti-GFP (1:250, Aves Labs) and rat anti-PDF (1:250; Depetris-Chauvin et al., 2011). The following secondary antibodies were used: Cy2-conjugated anti-chicken, Cy5-conjugated anti-rat, and Cy3-conjugated anti-rabbit (1:250, Jackson ImmunoResearch Laboratories, Inc). Images were acquired with a ZEISS LSM 880 Confocal Laser Scanning Microscope.

## Results

### Retina Photoreceptors Are Involved in Sleep Regulation

**FIGURE 1:**
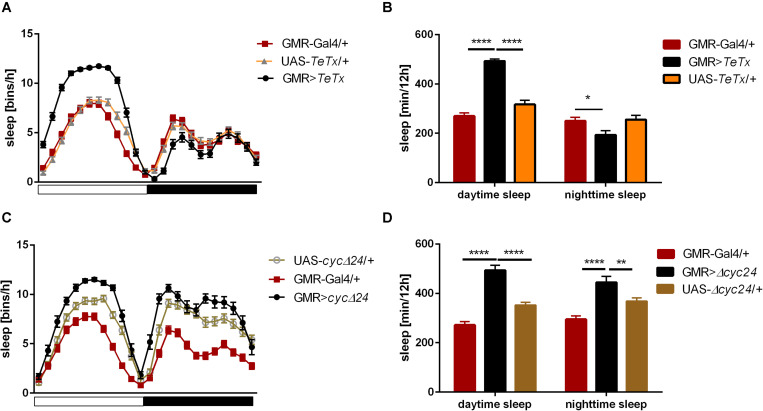
Effects of *glass*-expressing cells on sleep. **(A)** Sleep pattern of flies with blocked neurotransmission from photoreceptors (GMR>*TeTx*) measured as number of sleep bins per hour. **(B)** Total sleep time of GMR>*TeTx* flies measured as minutes per 12 h, separately for day and night-time. **(C)** Sleep pattern of flies with clock disruption in photoreceptors (GMR>Δ*cyc24*). **(D)** Total sleep time of GMR>Δ*cyc24* flies. Heterozygous parental strains were used as control. Statistically significant differences marked with asterisk *p ≤ 0.05; **p ≤ 0.01; ****p ≤ 0.0001. Detailed statistics are presented in Supplementary Tables S3, S4.

To analyze the contribution of specific photoreceptors to the regulation of rhythmic behavior we first examined the impact of blocking light input through the retina. In order to do so we took advantage of the GMR>*TeTx* strain, in which neurotransmission from cells expressing *glass* was totally blocked by expression of tetanus toxin light chain, which cleaves synaptobrevin protein and blocks neurotransmitter release. GMR>*TeTx* flies showed no defects in rhythmicity or periodicity, but the evening anticipation was increased (Table 1). Interestingly, these flies exhibited an altered sleep profile (Figure 1A) with increased sleep time during the day (Figure 1B), resulting in lower levels of total activity (Supplementary Figure S1A).

**TABLE 1:** Locomotor activity of flies with disrupted synaptic transmission (TeTx) or molecular clock (Δ*cyc24*) in the specific type of cells in the visual system.

Genotype	Period [h]	% Rhythmic	MAI	EAI	Number of flies
UAS-*cycΔ24*/+	23.7	93	0.63	0.76	88
UAS-*TeTx*/+	24.0	94	0.56	0.79	84
UAS-*ChATRNAi*/+	23.3	100	0.69	0.65	58
GMR-Gal4/+	23.9	99	0.54	0.74	92
GMR>Δcyc24	22.9****	45*	0.64	0.9****	91
GMR>*TeTx*	23.8	95	0.52	0.91****	91
*Rh1*-Gal4/+	23.3	100	0.58	0.8	91
*Rh1*>Δcyc24	23.7	77	0.7	0.79	92
*Rh1*>*TeTx*	23.5	100	0.79****	0.8	91
*Rh3*-Gal4/+	24.2	97	0.71	0.72	124
*Rh3*>Δ*cyc24*	23.9	83	0.57	0.72	71
*Rh3*>*TeTx*	24.1	75	0.73	0.7	84
*Rh5*-Gal4/+	24.3	95	0.63	0.81	95
*Rh5*>Δcyc	24.2	80	0.59	0.78	70
*Rh5*>*TeTx*	24.3	97	0.56	0.71	89
*Rh6*-Gal4/+	23.7	78	0.63	0.7	78
Rh6>Δcyc	23.6	99	0.59	0.91****	74
*Rh6*>*TeTx*	23.6	66	0.6	0.72	74
*Rh6*>*Val10*	23.4	96	0.58	0.78	55
*Rh6*>*ChatRNAi*	23.7	86	0.52	0.88*	73
L2>*TeTx*	24.0	96	0.65	0.87****	91
L2-Gal4/+	24.0	92	0.64	0.8	90

Columns present: genotype, period of locomotor activity, % of rhythmic flies, morning anticipation index (MAI), evening anticipation index (EAI), and total number of flies used for the experiments. Experimental flies which showed differences with both controls are highlighted with bold. Statistically significant differences marked with asterisks (*p ≤ 0.05; ****p ≤ 0.0001). Detailed statistics are presented in Supplementary Tables S1, S2.

The retina photoreceptors are peripheral oscillators with rhythmic expression of clock genes. To check whether retinal clocks are involved in the network regulating sleep in flies, we used GMR>Δ*cyc24* strain, in which expression of the dominant negative form of CYCLE causes disruption of the molecular clock in photoreceptors. We found that this manipulation triggered arrhythmicity in 55% of GMR>Δ*cyc24* flies in constant darkness conditions (Table 1) and in rhythmic flies, the period was shorter (22.9 h) compared to the control (Table 1). Moreover these flies exhibited an increased morning anticipation index (Table 1), altered sleep pattern and length during the day and night (Figures 1C,D), and a decreased total activity (Supplementary Figure S1A). Knowing that *cyc* dominant negative overexpression in *tim*-Gal4 cells is lethal (Chow et al., 2016), we carefully examined the eye morphology and anti-BRP labeling in the brain to exclude a possibility that the observed results originate from some neuronal degeneration. However, we did not observe any changes in the examined individuals.

These two experiments showed that *glass*-expressing cells, which are mostly retinal photoreceptors, are involved in the regulation of sleep, while they are not necessary to maintain the locomotor activity rhythm, as it has already been shown (Grima et al., 2004).

### The Clock Located in the Retina Photoreceptors Regulates Their Own Circadian Output

**FIGURE 2:**
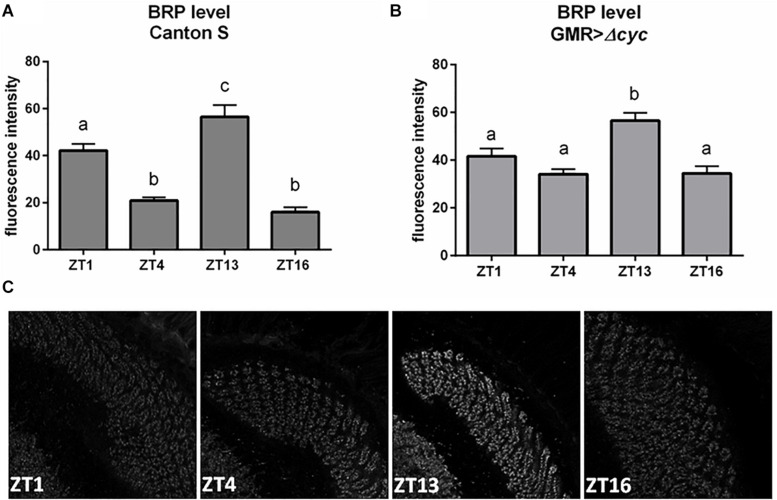
Effects of peripheral clocks located in the photoreceptors on the presynaptic protein Bruchpilot (BRP) expression. The immunofluorescence signal intensity was measured in the distal lamina on cryosections of control [Canton S, **(A)**] and experimental [GMR>Δ*cyc24*, **(B)**] flies at four time points (ZT1, ZT4, ZT13, and ZT16). Statistically significant differences were marked with letters, where different letters above the bar means confirmed changes between time points. **(C)** Confocal images of BRP immunostaining in the lamina of GMR>Δ*cyc24* at different time points.

To investigate how oscillators located in the retina transmit rhythmic signals to the deep brain, we looked at the effect of peripheral clock disruption on the presynaptic protein Bruchpilot (BRP) cycling in the photoreceptor terminals in the lamina. Under LD12:12 BRP levels oscillate daily with two maxima observed at the beginning of the day (ZT1) and at the beginning of the night (ZT13) (Figure 2A; Górska-Andrzejak et al., 2013). Interestingly, in our study in GMR>Δ*cyc24* flies the expression was changed, the BRP level at ZT1 was not significantly different than at ZT4 and ZT16, and only one peak at the beginning of the night (ZT13) was observed (Figures 2B,C). This result is in accordance to what was already described for DD conditions, reinforcing the notion that morning BRP peak is controlled by light (Górska-Andrzejak et al., 2013). However, the fluorescence intensity level at ZT1 in the experimental and control flies was similar, and enhanced at ZT4 and ZT16, which may suggest that BRP degradation rather than expression is affected.

**FIGURE 3:**
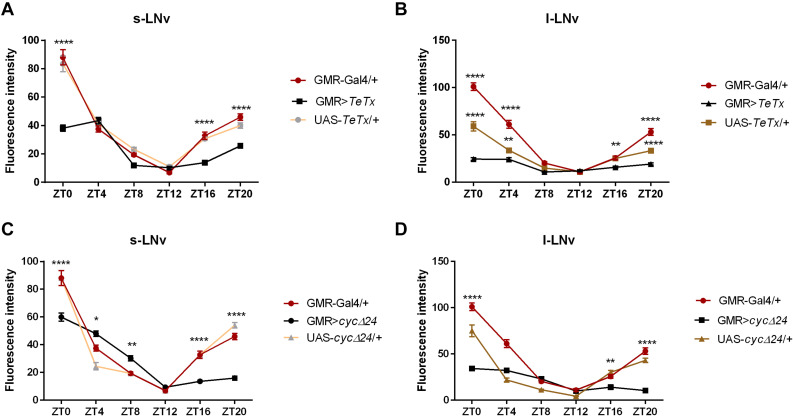
Effects of peripheral clock located in the photoreceptors on *per* expression in the pacemaker. The immunofluorescence signal intensity was measured in s-LN_*v*_s and l-LN_*v*_s marked with anti-PDF staining for GMR>*TeTx* **(A,B)**, and GMR>Δ*cyc24* **(C,D)** experimental flies. Asterisks (*p ≤ 0.05; **p ≤ 0.01; ****p ≤ 0.0001) show statistically significant differences between experimental flies and controls at specific time point. Detailed statistics are presented in Supplementary Table S5.

We then explored the effect of peripheral clock located in the photoreceptors on the pace of the main oscillator, by blocking neurotransmission from the retina photoreceptors (GMR>*TeTx*) or disrupting the clock in these cells (GMR>Δ*cyc24)*. Strikingly, we found that GMR>*TeTx* and GMR>Δ*cyc24* flies display a clear dampening of PER oscillations in the small and large LN_*v*_s (Figures 3A–D, respectively). Thus, blocking neurotransmission from the retina photoreceptors and disruption of the clock in *glass*-expressing cells decreased the amplitude in PER cycling in essential pacemaker neurons (Figure 3).

### Specific Photoreceptor Types Regulate Sleep at Different Ways

Because our experiments with GMR strain showed changes in sleep pattern and level, we focused on this behavior in the next experiments. GMR expression is not limited to the retina, however. In fact, GMR is expressed in some clock neurons (DN1p; Klarsfeld et al., 2004), which are involved in the sleep regulation (Guo et al., 2018; Lamaze and Stanewsky, 2020). To investigate which cell types triggered the observed responses, we expressed tetanus toxin in different types of photoreceptors, using various rhodopsin drivers, which allowed us to exclude the effect of DN1p. Surprisingly, we obtained different effects depending on photoreceptor types. In general, blocking input from different photoreceptor types resulted in decreased total activity, but their effects on sleep and morning/evening peaks were different. No changes in the period of locomotor activity were observed (Table 1).

**FIGURE 4:**
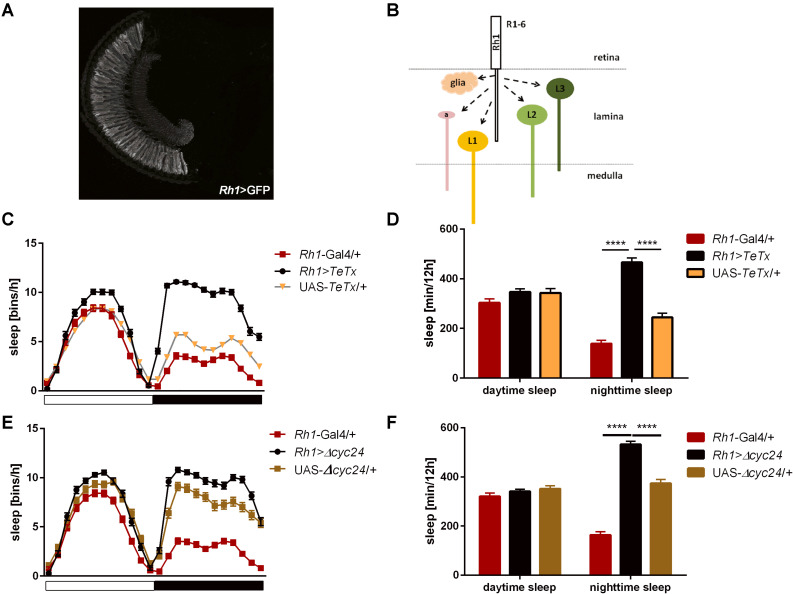
R1-6 photoreceptors control sleep during the night. **(A)** *Rh1*-expressing cells are R1-6 photoreceptors, with terminals in the lamina (cryosection of *Rh1*>GFP brain). **(B)** Graphical presentation of pathways which are blocked after TeTx expression in *Rh1*-expressing cells (a – amacrine cells, L1-3 – lamina monopolar cells). **(C)** Sleep pattern of *Rh1>TeTx* flies. **(D)** Total sleep amount during the day and night after blocking of synaptic transmission from R1-6 (*Rh1>TeTx*). **(E)** Sleep pattern for *Rh1>Δcyc24* flies. **(F)** Total sleep time of *Rh1>Δcyc24* strain. Heterozygous parental strains were used as control. Statistically significant differences marked with asterisks ****p ≤ 0.0001. Detailed statistics are presented in Supplementary Table S3.

After blocking light transmission pathway from R1–R6 cells (*Rh1*-expressing; Figures 4A,B) we observed clear changes in the sleep pattern (Figure 4C) with significant longer sleep time during the night, no effect on day-time sleep (Figure 4D), and a subtly reduced evening peak (Table 2 and Supplementary Figure S2A). These effects were opposite to those observed in case of GMR; nevertheless, in this case a decrease of total activity was also observed (Supplementary Figure S1B).

**TABLE 2:** Morning and evening peaks of activity of flies with disrupted synaptic signaling (TeTx) or molecular clock in specific type of cells in the visual system compared with parental strains (Gal4/+ and UAS/+, respectively).

	Morning peak						Evening peak					
		GAL4/+	*p*-value	UAS/+	*p*-value	*F* (DFn, dFD)		Gal4/+	*p*-value	UAS/+	*p*-value	*F* (DFn, dFD)
*Rh1*>*TeTx*	83.8	76.4	0.271	84.0	0.9985	2.080(2,384)	**100.2**	183.8	< 0.0001	134.5	0.0007	45.68(2,384)
*Rh1*>*cycΔ24*	69.4	76.4	0.3044	85.9	0.015	3.93(2,281)	112.6	183.8	< 0.0001	129.9	0.1753	37.43(2,360)
*Rh3*>*TeTx*	**98.4**	79.2	0.0011	84.0	0.0171	6.576(2,341)	**77.0**	111.1	0.0002	134.5	< 0.0001	24.51(2,341)
*Rh3*>*cycΔ24*	**105.2**	79.2	< 0.0001	85.9	0.0036	13.38(2,242)	113.6	111.1	0.9576	129.9	0.1486	4.077(2,321)
*Rh5*>*TeTx*	**64.2**	83.8	0.0004	84.0	0.0001	11.05(2,396)	**81.6**	116.8	< 0.0001	134.5	< 0.0001	31.04(2,396)
*Rh5*>*cycΔ24*	**58.7**	83.8	0.0002	85.9	0.0007	9.931(2,239)	**86.7**	116.8	0.001	129.9	< 0.0001	14.51(2,318)
*Rh6*>*Tetx*	93.9	72.1	0.0004	84.0	0.1361	7.514(2,330)	101.7	102.7	0.9928	134.5	0.0002	11.84(2,330)
*Rh6*>*cycΔ24*	84.3	72.1	0.0603	85.9	0.9663	3.904(2,224)	131.9	102.7	0.0029	129.9	0.9677	8.013(2,303)
*L2*>*TeTx*	**59.3**	75.7	0.0014	84.0	< 0.0001	15.05(2,360)	113.3	111.3	0.9647	134.5	0.0157	6.635(2,360)
*Rh6*>*ChaTRNAi*	51.9	87.0	< 0.0001	52.2	0.9991	25.27(2,247)	**109.3**	84.9	0.0011	39.6	< 0.0001	49.43(2,247)

*Experimental flies which showed statistically significant differences with both controls are highlighted with bold. Each experimental strain was compared with control strains (Gal4 and UAS) using one-way ANOVA and Tukey’s test. Genotypes with *p* < 0.05 with both controls are marked as statistically significant changes with bold. Degrees of freedom [*F* (DFn, DFd)] are listed for every group.*

Similar effects were obtained after disrupting the clock in the R1-6 photoreceptors (Figure 4E), despite the impact on night-time sleep was less pronounced (Figure 4F and Supplementary Figure S3A).

**FIGURE 5:**
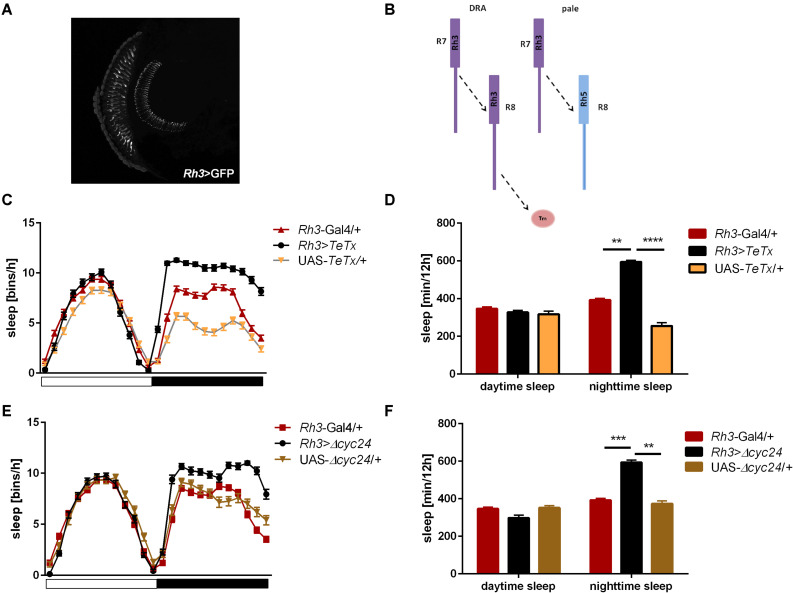
*Rh3*-expressing cells affect night-time sleep. **(A)** Rh3 is expressed in R7 and R8 cells, which terminate in the medulla (cryosection of *Rh3*>GFP brain). **(B)** Graphical presentation of synaptic connections formed by *Rh3*-expressing cells which are blocked in *Rh3>TeTx* flies (Tm – transmedulla neurons). **(C)** Sleep pattern of *Rh3>TeTx* flies. **(D)** Sleep time during the day and night of *Rh3>TeTx* flies. **(E)** Sleep pattern of *Rh3>Δcyc24.* **(F)** Amount of sleep during the day and night of *Rh3>Δcyc24.* Heterozygous parental strains were used as control. Statistically significant differences marked with asterisks **p ≤ 0.01; ***p ≤ 0.001; ****p ≤ 0.0001. Detailed statistics are presented in Supplementary Table S3.

We next inquired the relevance of the R7-8 photoreceptors. The block of neurotransmission from the Rh3-expressing cells, reaching the medulla (Figures 5A,B), had strong effect on the activity patterns, with an increased morning peak and a decreased evening peak (Table 2 and Supplementary Figure S2B). The sleep pattern was also affected (Figure 5C), with increased night-time sleep (Figure 5D), and decreased total activity (Supplementary Figure S1C). Similarly, the clock disruption in these cells resulted in a decreased total activity, although the impact on night-time sleep was less pronounced (Figures 5E,F and Supplementary Figures S1C, S3B).

**FIGURE 6:**
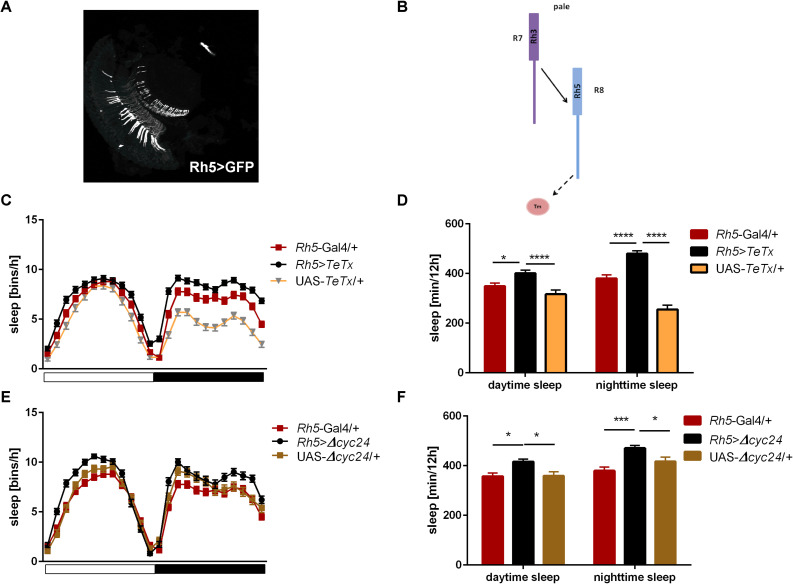
*Rh5*-expressing cells affect sleep both, during the day and night. **(A)** Rh5 is expressed in R8 cell which terminates in the medulla (cryosection of *Rh5*>GFP brain). **(B)** Graphical representation of pathways blocked in *Rh5>TeTx* strain. Solid line represents input to R8 cell coming from R7. Dashed line represents blocked output from R8 to downstream cells. **(C)** Sleep pattern of *Rh5>TeTx.* **(D)** Sleep time during the day and night of *Rh5>TeTx* flies. **(E)** Sleep pattern of *Rh5>Δcyc24.* **(F)** Amount of sleep during the day and night of *Rh5>Δcyc24.* Heterozygous parental strains were used as control. Statistically significant differences marked with asterisks *p ≤ 0.05; ***p ≤ 0.001; ****p ≤ 0.0001). Detailed statistics are presented in Supplementary Table S3.

Next we examined the contribution of Rh5-expressing R8 “pale” ommatidia (Figures 6A,B). *Rh5*>*TeTx* flies exhibited reduced total activity (Supplementary Figure S1D) and both morning and evening activity peaks were decreased compared to controls (Table 2 and Supplementary Figure S2C). The sleep pattern changed (Figure 6C) with increased sleep time in both, day and night (Figure 6D). Overexpression of *cycΔ24* in “pale” R8 cells recreated the effects linked to TeTx expression: decreased total activity (Supplementary Figure S1D), blunted morning and evening peaks (Table 2 and Supplementary Figure S3C) and changes in the sleep pattern (Figure 6E) and level during the day and night (Figure 6F).

**FIGURE 7:**
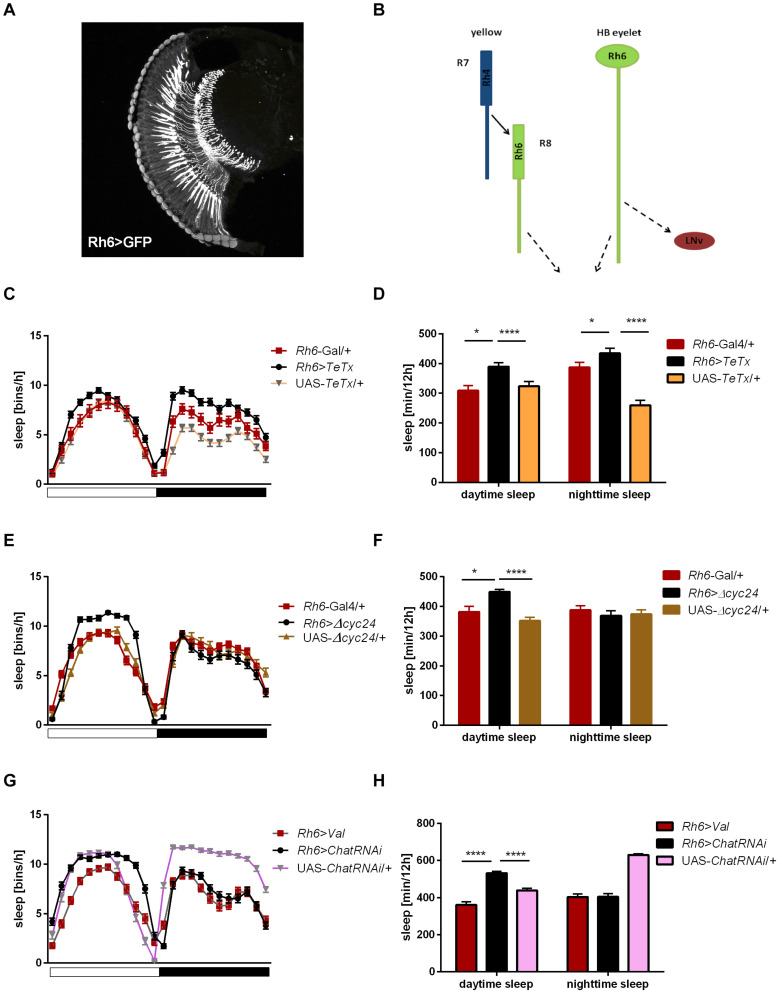
*Rh6*-expressing cells regulate sleep during the day and night. **(A)** *Rh6*-expressing photoreceptors (R8) terminate in the medulla. The cryosection of *Rh6*>GFP brain does not show Hofbauer-Buchner (HB) eyelets. **(B)** Graphical presentation of synaptic contacts (dotted line) between *Rh6*-expressing photoreceptors or HB eyelets and their targets. Solid line represent input to R8 coming from R7 cell. **(C)** Sleep pattern of *Rh6>TeTx* flies. **(D)** Sleep time of *Rh6>TeTx* flies is increased during the day and night. **(E)** Sleep pattern of *Rh6>Δcyc24.* **(F)** Sleep amount of *Rh6>Δcyc24*. **(G)** Sleep pattern of flies with downregulated acetylcholine synthesis in HB eyelets (*Rh6>ChAT-RNAi*). **(H)** Sleep amount of *Rh6>ChAT-RNAi* is increased during the day only. Heterozygous parental strains were used as control, additional control *Rh6>Valium10-GFP* was used for the last experiment. Statistically significant differences marked with asterisks *p ≤ 0.05; ****p ≤ 0.0001. Detailed statistics are presented in Supplementary Table S3.

Rh6 is expressed in the R8 “yellow” ommatidia as well as in the HB eyelets (Figures 7A,B). Blocking the synaptic transmission from these cells did not affect overall activity levels (Supplementary Figures S1E, S2D) and the sleep was slightly extended at the beginning of the day and during the night (Figures 7C,D). Clock disruption in this type of photoreceptors only mirrored the one triggered during the day (Figures 7E,F and Supplementary Figure S3D).

To distinguish between the contribution of R8 photoreceptors and the HB eyelets, both expressing Rh6, we knocked down the expression of choline acetyltransferase, necessary for the synthesis of acetylcholine, used as neurotransmitter by the HB eyelets. In *Rh6*>*ChATRNAi* flies changes in sleep pattern and increased sleep time became evident only during the day (Figures 7G,H).

Taking these data together, we can conclude that R1-6 cells, as well as Rh3 and Rh6-expressing retinal photoreceptors affect sleep during the night, while R8 expressing Rh5 is involved in both day- and night-time sleep regulation. Moreover, cholinergic HB eyelets play a wake-promoting role during the day.

### L2 Interneurons as an Additional Peripheral Clock Output

**FIGURE 8:**
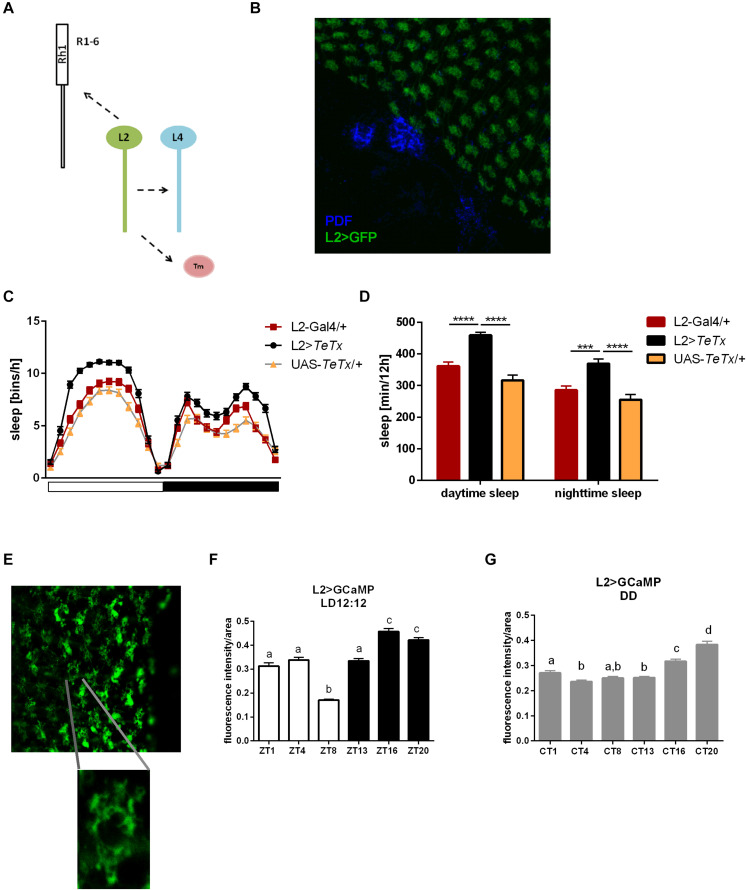
L2 interneurons play important role in the regulation of sleep. **(A)** Graphical presentation of synaptic contacts formed by L2 (dotted line) (Tm –Transmedulla neurons). **(B)** L2 terminals are located in close proximity to PDF-immunoreactive LN_*v*_s neurons (whole mount immunostaining of L2>GFP flies with anti-GFP and anti-PDF antibodies, blue). **(C)** Sleep pattern of L2>*TeTx* flies. **(D)** Sleep amount presented for flies with tetanus toxin expression in L2 cells. Statistically significant differences marked with asterisks ***p < 0.001; ****p < 0.0001. **(E)** Fluorescence calcium indicator expressed in L2 cells was measured in the terminals in the medulla. **(F)** Calcium level in the L2 terminals measured in flies kept in LD12:12 conditions. **(G)** Calcium level in the L2 terminals of flies in constant darkness (DD). Statistically significant differences were marked with letters, where different letters above the bar means confirmed changes between time points.

Since light signals are transmitted to the pacemaker neurons from the retina photoreceptors via the lamina interneurons, we examined the behavior of flies with blocked neurotransmission in L2 interneurons, which receive light input from R1–R6 cells (Figure 8A). We focused on this cell type because it shows rhythmic changes in the size of dendritic trees,’ probably connected with daily changes in signal transmission. Moreover, L2 terminals are located in a close vicinity to l-LN_*v*_s (Figure 8B). These flies displayed behavioral changes reminiscent of GMR>*TeTx* flies, with a smaller morning peak of activity (Table 2). The sleep pattern of these flies was changed (Figure 8C) with increased sleep duration during the day and night (Figure 8D).

To correlate daily changes in the size of dendritic trees’ with activity of L2 cells, we carried out calcium imaging using L2>*GCaMP6f* transgenic strain. GCaMP6f is a calcium indicator, which allows to measure Ca^2+^ level correlated to the indicator fluorescence intensity. In our experiment we isolated brains at selected time points and measured the fluorescent signal immediately after dissection. The fluorescent intensity in the L2 terminals was measured in the medulla (Figure 8E) and calculated per terminal area, comparing Ca^2+^ level/area unit time points. In LD12:12, Ca^2+^ level was highest at night (ZT16, ZT20) and lowest in the middle of the day (ZT8; Figure 8F). In DD this pattern subtly changed, with the highest intensity signal at CT16 and CT20, but without the lowest level at ZT8 (Figure 8G).

The obtained results suggest that L2 interneurons play important wake-promoting role. It is supported by the fact that calcium levels in the terminals are the lowest during siesta.

### Photic Inputs to the Pacemaker May Be Regulated by Synaptic Plasticity

**FIGURE 9:**
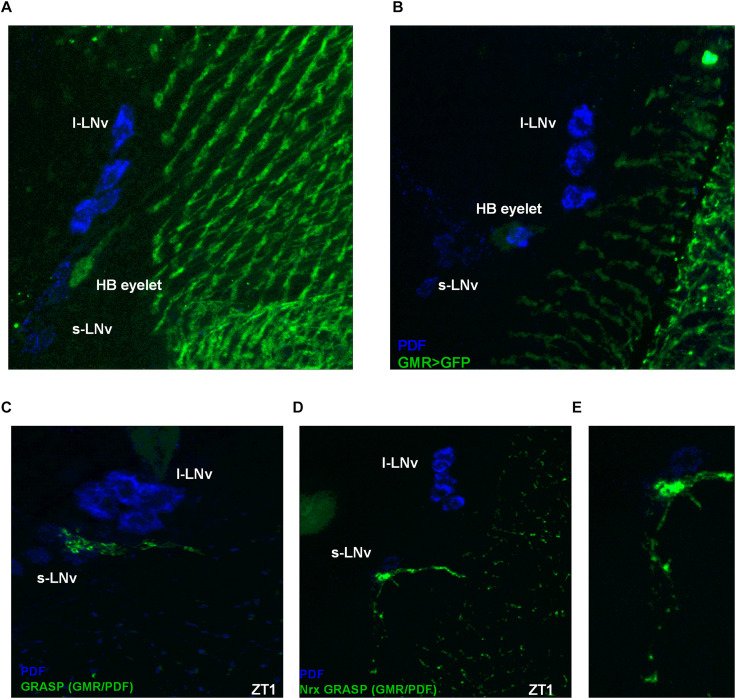
Hofbauer-Buchner eyelets contact with LN_*v*_s. **(A,B)** Immunostaining of GMR>GFP flies shows that HB terminals are located in aMe area, in close proximity to s-LN_*v*_s. **(C)** GFP reconstitution across synaptic partners (GRASP) technique allows to visualize synaptic contacts between HB eyelets and PDF-expressing cells, according to location identified as s-LN_*v*_s. **(D,E)** Nrx GRASP technique confirmed that HB form active synaptic contacts with s-LN_*v*_s.

Some photoreceptor terminals are located next to the clock neurons in a region called the accessory medulla (Figures 9A,B). It was previously shown that the visual system can directly communicate with clock neurons and receive photic information through the HB eyelets (Muraro and Ceriani, 2015; Schlichting et al., 2016). Taking advantage of the GFP reconstitution across synaptic partners (GRASP) technique (Feinberg et al., 2008) we found that the HB eyelets terminals differentially contact the LN_*v*_s during the day, with stronger contacts during light phase (Figure 9C). To confirm that these results correlate with differential synaptic connectivity, we used Nrx GRASP to visualize only active synaptic contacts (Figures 9D,E). At ZT1 100% of brains showed reconstituted signal (*n* = 20), the proportion of brains decreased at other time points, i.e., 43% at ZT4 (*n* = 21), 59% at ZT13 (*n* = 22), and 35% at ZT16 (*n* = 20), suggesting that the LN_*v*_s receive direct input from the HB eyelets in a plastic manner, preferentially during the early morning.

The HB eyelets terminals arborize in a close vicinity to the s-LN_*v*_s cell bodies. Taking into account the localization of the reconstituted signal, it is likely the HB eyelets communicate with the s-LN_*v*_s rather than the l-LN_*v*_s.

**FIGURE 10:**
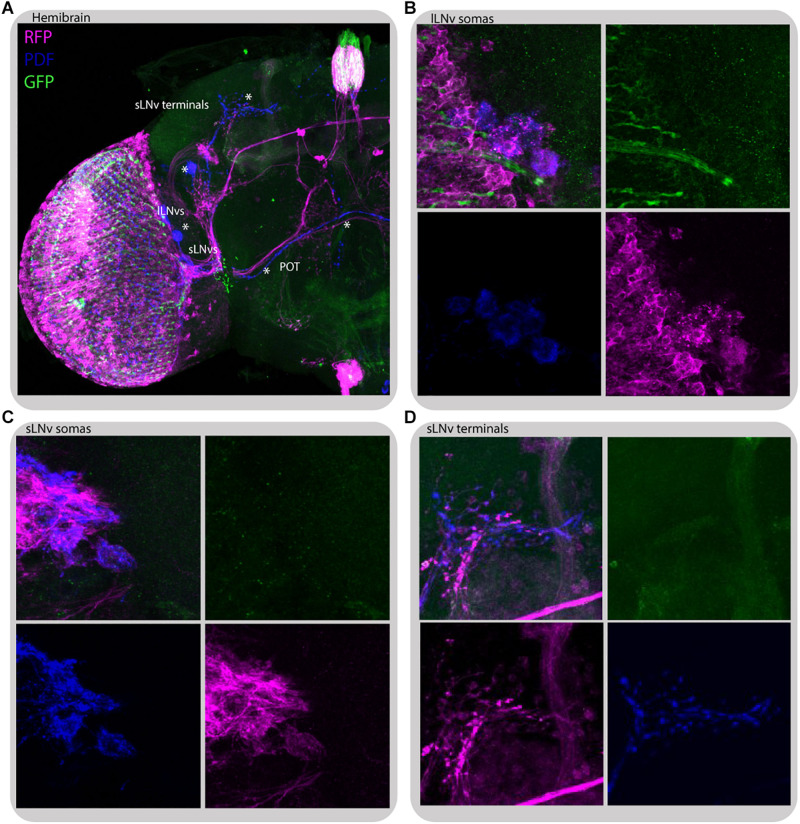
*Glass*-expressing cells contact both l-LN_*v*_s and s-LN_*v*_s. Immunostaining of GMR>*transTANGO* adult male brain. *transTANGO* marks presynaptic *glass*-expressing cells with GFP and the postsynaptic partners of those cells with RFP. Anti-PDF immunostaining confirms that both small and large LN_*v*_s are postsynaptic to GMR-Gal4 recruited cells. **(A)** Hemibrain. Asterisks were placed where PDF and RFP labeling co-localize: in posterior optic tract (POT) coming from l-LN_*v*_s, s-LN_*v*_s, and their terminals in the dorsal brain. **(B)** l-LN_*v*_s somatas (labeled with PDF, blue) have postsynaptic mark (RFP, magenta), HB eyelets projections can be seen nearby (labeled with GFP, green). **(C,D)** s-LN_*v*_s somatas and terminals have postsynaptic mark (RFP, magenta). Every brain analyzed (*n* = 9) showed similar staining. The images were acquired from different brains.

**FIGURE 11:**
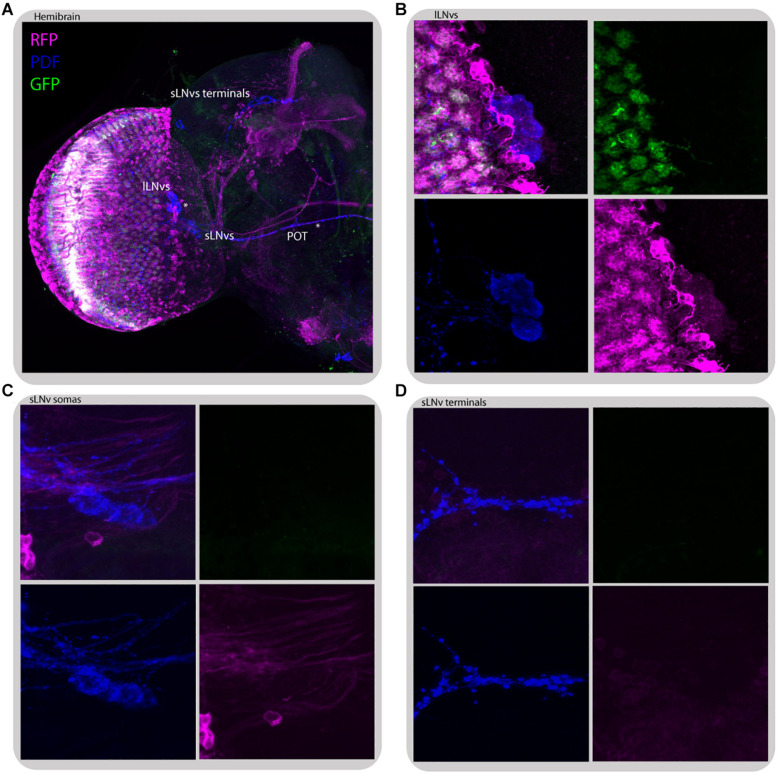
L2 interneurons form direct synaptic contacts with large but not small LN_*v*_s. Immunostaining of L2>*transTANGO* adult male brain. **(A)** Hemibrain. Asterisks mark places where PDF and RFP labeling co-localize, which means l-LN_*v*_s cell bodies and POT. **(B)** l-LN_*v*_s somatas have postsynaptic labeling, meaning that l-LN_*v*_s form direct contacts with L2 interneurons. This was seen in 86% of brains (*n* = 7). A L2 projection can be seen in cyan, rounding a somata. **(C,D)** Neither somatas nor projections of the s-LN_*v*_s have postsynaptic labeling. None of the brains analyzed showed RFP signal in these cells. The images were acquired from different brains.

To gain insight into how the information from peripheral oscillators is transmitted to the main clock we took advantage of *transTANGO* (Talay et al., 2017) to uncover postsynaptic cells to photoreceptors (GMR-Gal4) and L2 interneurons (L2-Gal4; Figures 10, 11, respectively). This tool uses a modified signaling pathway to express RFP in the postsynaptic cells of a GAL4 of interest, while marking the cells recruited by that driver with GFP. In pursuit of the postsynaptic targets to GMR+ photoreceptors we observed red fluorescence in the lamina and medulla, probably coming from the lamina interneurons and amacrine cells. The signal was also detected in a few cell bodies in the accessory medulla, which project to the dorsal brain and to the contralateral side of the brain. Double staining with antibodies against PDF confirmed that a subset of RFP-expressing cells belong to both the s-LN_*v*_ and l-LN_*v*_ groups and to processes described as the posterior optic tract (POT), which connect the l-LN_*v*_ cluster located on both sides of the brain, and dorsal projections of the s-LN_*v*_s. Closer inspection of the GFP signal indicated that the cells contacting LN_*v*_s are those of the HB eyelets (Figure 10C), confirming what is depicted in the recently published connectome (Scheffer et al., 2020).

Despite no direct contact between L2 neurons and LN_*v*_s was detected through GRASP (Muraro and Ceriani, 2015), *transTANGO* mapping of L2 postsynaptic targets highlighted the l-LN_*v*_s, but not the s-LN_*v*_s, in the majority of brains (86% positive, *n* = 7) (Figure 11). This l-LN_*v*_s exclusive connection could help explain why disrupting the connectivity or the clock in the retinal peripheral oscillator has a stronger effect on the sleep pattern than in other circadian outputs examined.

## Discussion

The circadian clock relies on a self-sustained molecular mechanism, which is entrained to the daily changes of environmental conditions. The most powerful factor synchronizing circadian clocks is light. The LN_*v*_s, essential circadian pacemakers, receive light information through the visual system and the deep brain photoreceptor CRY (Emery et al., 2000; Sheeba et al., 2008a).

The involvement of the visual system is still not completely understood. Pacemaker cells are responsible for the temporal organization of rhythmic behavior, such as locomotor activity and sleep but also contribute to other features such as sleep length during the day and night and total activity level (Parisky et al., 2008; Shang et al., 2008; Chung et al., 2009; Potdar and Sheeba, 2018). In this study we focused on the role of retinal and extra-retinal photoreceptors in the transduction of photic information to modulate sleep patterns. We uncovered that peripheral clocks in the eye contribute to maintaining sleep pattern with little effects on the period of locomotor activity rhythm, as expected (Grima et al., 2004).

Clock disruption in *glass*-expressing cells triggered arrhythmicity, and in the remaining rhythmic flies, a shorter period of the locomotor activity pattern. Surprisingly, blocking synaptic transmission from photoreceptors to postsynaptic cells add no effects on either rhythmicity or periodicity, however, observed changes in the sleep pattern and level were similar in both experiments. Subsets of clock neurons employ CRY for light entrainment, while the others use CUL-3 mediated mechanism for molecular light resetting (Ogueta et al., 2020). However, it seems that they need rhythmic inputs from the visual system, as arrhythmic signaling may affect the molecular mechanism of pacemaker neurons.

The visual system shows daily changes of sensitivity to light. In addition, the clock-controlled optomotor response in *Drosophila* is higher at night than during the day (Mazzotta et al., 2013; Damulewicz et al., 2017). In the first neuropil (lamina) of the optic lobe, circadian rhythms have been observed at the cellular (Weber et al., 2009) and molecular levels (Górska-Andrzejak et al., 2013) including the expression of several genes and proteins, for example the alpha subunit of the sodium/potassium pump in the lamina glia (Górska-Andrzejak et al., 2009; Damulewicz et al., 2013).

In the present study, we showed that not only sensitivity of photoreceptors, but also interneurons involved in light transmission changes during the day. L2 monopolar cells, one of the postsynaptic cells in tetrad synapses, which hyperpolarize in response to light, show daily rhythms in calcium levels. The highest Ca^2+^ concentration in the terminals was observed at night, similarly to the retina photoreceptors. It seems that the visual system is more sensitive to light during the dark phase to detect low intensity light, such as moonlight, and such sensitivity decreases at the end of the night to be prepared for high intensity of light in the morning (Shang et al., 2008; Sheeba et al., 2008b; Nippe et al., 2017).

We showed that rhythms observed in the lamina, like the presynaptic protein BRP expression, is controlled by both the pacemaker and the oscillators located in the retina. Moreover, the BRP expression pattern in flies in which the peripheral clock in the retina is disrupted, is unimodal, similar to that observed in constant darkness, when the morning peak of BRP is missing and only the evening peak is observed (Górska-Andrzejak et al., 2013). However, in our experimental model BRP level at ZT1 was similar to those observed in control, which suggests that clock disruption affects rather degradation of BRP than its expression.

Despite the strong effect on sleep of genetic manipulations in photoreceptors using the GMR-Gal4 driver, the *glass* gene is also expressed in some DN_1__*s*_ and in a group of cells located next to LN_*d*_s (Vosshall and Young, 1995; Klarsfeld et al., 2004), opening the possibility that the results obtained could not be exclusively eye-specific (Li et al., 2012; Ray and Lakhotia, 2015). To avoid multi-cellular effects, we focused on data performed with drivers specific to different photoreceptor types, and we observed various effects on sleep, depending on the photoreceptor type.

The previous analysis employing the *norpA* mutant, which lacks phospholipase C in the canonical phototransduction pathway (Pearn et al., 1996), showed that light signals to the clock are transmitted not exclusively from the retina photoreceptors, but also by the circadian photoreceptor CRY and the HB eyelets (Emery et al., 2000; Helfrich-Förster et al., 2001). However, *norpA* mutants are not able to entrain to changes in light conditions, because of reduced circadian sensitivity to light (Emery et al., 2000). Moreover, in the double mutant *norpA; cry^*b*^*, PER cycling was maintained in s-LN_*v*_s and DN_1_s but abolished in l-LN_*v*_s and LN_*d*_s, suggesting that these clusters are photoentrained by an alternative pathway (Helfrich-Förster et al., 2001). This conclusion is further supported by the fact, that an alternative *norpA*-independent phototransduction pathway occurs in Rh1, Rh5, and Rh6-expressing photoreceptors (Shortridge et al., 1991; Szular et al., 2012; Ogueta et al., 2018). Flies with blocked synaptic transmission using GMR>*TeTx* resembled the *norpA* mutant. Since this genetic combination blocks synaptic transmission between photoreceptors, R1–R8, the HB eyelets, and their postsynaptic partners, the effect on sleep pattern was stronger compare to that in *norpA* mutants. More severe changes in PER expression were observed in l-LN_*v*_s than s-LN_*v*_s after impairing input from the photoreceptor cells, which is consistent with previous reports (Helfrich-Förster et al., 2001).

Lack of photic information from R1–R6 increased sleep duration at night. However, blocking synaptic transmission from L2 interneurons caused more complex effects on both day- and night-time sleep. L2 interneurons are important for front-to-back motion detection at intermediate pattern contrast (Rister et al., 2007). They are postsynaptic to R1–R6 photoreceptors and to L4 interneurons and presynaptic in feedback synapses, which are formed back to them (both to R1-6 and L4) and use acetylcholine and glutamate as neurotransmitters (Meinertzhagen and O’Neil, 1991; Kolodziejczyk et al., 2008; Raghu and Borst, 2011; Takemura et al., 2011, 2015; Hu et al., 2015). These feedback synapses play a role in preventing photoreceptors from saturation and improving signal quality (Zheng et al., 2006), however, they seem to be extremely important for the functioning of R1–R6 photoreceptors. R1-6 transmit signal to different types of cells, which belongs to pathways specialized to detect contrast increments (ON pathway, L1) or decrements (OFF pathway, L2, L3; Joesch et al., 2010). In effect, blocking R1-6 signaling affects both pathways, however, L2 cells can still receive signal from L4, and send information to deeper parts of the brain. On the other hand, lack of neurotransmission from L2 cells can disrupt not only downstream OFF pathway, but also proper R1-6 functioning, giving the effect on both, day- and night-time sleep. Moreover, according to our *transTANGO* data, L2 can also directly contact the l-LN_*v*_s. It is possible, however, that these synapses are formed only in a specific time of the day, or contacts are very weak and conventional GRASP is not strong enough to show positive results (Muraro and Ceriani, 2015). This aspect needs to be addressed by more detailed experiments in the future.

Surprisingly, the lack of synaptic transmission from Rh3-expressing cells affected the morning peak of activity in a very specific way, opposite to that observed after blocking signaling from Rh5. Rhodopsin 3 is expressed in R7 in “pale” ommatidia and in both, R7 and R8, in the DRA type. Since the block of transmission from “pale” ommatidia, with R8 expressing Rh5, decreased the morning peak of activity, it is possible that Rh3, which absorbs UV, inhibits morning activity, while blue light, which is absorbed by Rh5, enhances the morning activity. This can also be an effect of the disruption in polarized light detection, which is received by DRA only.

Siesta or day-time sleep seems to be mostly regulated by R8 “pale” type of photoreceptors. The results obtained in this work may support the idea that blue light inhibits sleep during the day. The exposure to blue light seems to affect lifespan and induce neurodegeneration (Nash et al., 2019), and short sleep during the day allow avoiding UV and blue light during the day, thus protecting flies against harmful light exposure.

Rh6-expressing photoreceptors affect sleep in a specific way, since nap was longer after clock disruption but sleep during the night and day was increased after blocking transmission. Rh6, however, is expressed not only in the retina photoreceptors, but also in the HB eyelets. Our data suggest that R8 cells are involved in night-time sleep and HB eyelets in day-time sleep regulation. Acetylcholine expression silenced in Rh6-expressing cells increased sleep time only during the day, while tetanus toxin expression affected both day and night sleep time. As it is known that among Rh6-expressing cells only HB eyelets use acetylcholine as neurotransmitter we can conclude that observed effect is specific for these extracellular photoreceptors. It has been reported that the HB eyelets form direct synaptic contacts with pacemaker cells located in the aMe (Muraro and Ceriani, 2015; Li et al., 2018; Schlichting et al., 2019). Our GRASP results suggest that the s-LN_*v*_s are main target cells, however, it has been shown that also l-LN_*v*_s, ITP-expressing LN_*d*_s, DN_1a_ and DN_3a_ cells receive signals from the HB eyelets through terminals located in the aMe (Li et al., 2018), which was confirmed by *transTANGO* data. These contacts are the strongest at the beginning of the day, indicating that signaling between the HB eyelets and LN_*v*_s takes place in the morning. Down-regulation of histamine receptor expression in the pacemaker PDF-immunoreactive cells (*Pdf*>*HisCl*) results in lack of the morning anticipation, which is typical of clock mutants. In turn disruption of histamine signaling from photoreceptors to pacemaker cells affects sleep by increasing its length during the day and night (Oh et al., 2013). This means that histaminergic neurotransmission from photoreceptors is involved in the regulation of LN_*v*_s activity. It has been shown that the HB eyelets express histamine and acetylcholine (Pollack and Hofbauer, 1991; Yasuyama and Meinertzhagen, 1999; Yasuyama and Salvaterra, 1999) and LN_*v*_s have receptors for both neurotransmitters (McCarthy et al., 2011; Lelito and Shafer, 2012). According to our Nrx GRASP data the HB eyelets communicate with the s-LN_*v*_s directly at the beginning of the day. Schlichting et al. (2016) suggest that HB eyelets may also contact the l-LN_*v*_s, as histamine bath had inhibitory effect on l-LN_*v*_s (Schlichting et al., 2016), nevertheless, there are also other histamine sources in the aMe (Hamasaka and Nassel, 2006). Light signal received by HB eyelets in the morning is transmitted via acetylcholine and excite s-LN_*v*_s via nicotinic receptors (Wegener et al., 2004; McCarthy et al., 2011; Schlichting et al., 2016), causing increased cAMP level (Lelito and Shafer, 2012) and wake-promoting effect. This supports our results, which showed that down-regulation of acetylcholine expression in HB eyelets increases sleep time during the day.

Taking together, R1-6, R7 (Rh3-expressing photoreceptor), and R8 “yellow” (Rh6-expressing cells) seem to be important for night-time sleep regulation, and HB eyelets for day-time sleep, while the strongest effect, both during the day and night, has neurotransmission from R8 “pale” (Rh5-expressing cells). We observed a similar effect on night-time sleep when synaptic transmission was blocked from all types of photoreceptors, which means that during the night even weak light inputs received by retinal cells are important to keep flies awake. As a result of the decreased photoreception flies are less active and spend more time sleeping during the night. However, it was previously shown that the retinal photoreceptors play a role of peripheral oscillators and regulate daily changes in the visual system, we found that those oscillators also affect behavior. Disruption of the clock in a single type of photoreceptors decreased total activity, and in most cases, it affected sleep time and both morning and evening peaks of activity. These effects were similar to those observed after blocking transmission from specific photoreceptor types. This suggests that synaptic transmission is regulated by the photoreceptor clocks and light. In addition, we showed that HB eyelets, but also L2 interneurons can directly communicate with LN_*v*_s cells, which provides new light pathway to the clock neurons.

## Data Availability Statement

The raw data supporting the conclusions of this article will be made available by the authors, without undue reservation.

## Author Contributions

MD designed the study and wrote the manuscript with input from all co-authors. MD and JI performed the experiments and analyzed the data. All authors contributed to the article and approved the submitted version.

## Conflict of Interest

The authors declare that the research was conducted in the absence of any commercial or financial relationships that could be construed as a potential conflict of interest.

## Supplementary Material

FIGURE S1 Effects of blocking neurotransmission on activity pattern, performed for: **(A)** R1–R6 photoreceptors (Rh1), **(B)** Rh3-expressing cells, **(C)** Rh5-expressing cells, **(D)** Rh6-expressing cells, **(E)** L2 cells. Red arrows indicate statistically significant changes in morning or evening peak level. Detailed statistics is presented in Table 2.

FIGURE S2 Effects of clock disruption on activity pattern, performed for: **(A)** R1–R6 photoreceptors (Rh1), **(B)** Rh3-expressing cells, **(C)** Rh5-expressing cells, **(D)** Rh6-expressing cells. Red arrows indicate statistically significant changes in morning or evening peak level. Detailed statistics is presented in Table 2.

FIGURE S3 Effects of neurotransmission block (TeTx) or disruption of the peripheral clock on total activity level, performed for **(A)** *glass*-expressing cells (GMR), **(B)** R1–R6 photoreceptors (Rh1), **(C)** Rh3-expressing cells, **(D)** Rh5-expressing cells, **(E)** Rh6-expressing cells, **(F)** L2 interneurons. Heterozygous parental strains were used as control. Statistically significant differences marked with asterisks ^∗^*p* ≤ 0.05; ^∗∗^*p* ≤ 0.01; ^∗∗∗^*p* ≤ 0.001; ^****^*p* ≤ 0.0001). Detailed statistics is presented in Supplementary Table S4.

TABLE S1 Statistical analysis of the period of locomotor activity rhythm and percent of rhythmic flies. Each experimental strain was compared with control strains (Gal4/+ and UAS/+) using one-way ANOVA and Tukey’s test (for period) or non-parametric Kruskal–Wallis (for % rhythmic). Genotypes with *p* < 0.05 with both controls are marked as statistically significant changes with bold. Degrees of freedom [*F* (DFn, DFd)] are listed for every group.

TABLE S2 Statistical analysis of morning anticipation index (MAI) and evening anticipation index (EAI). Each experimental strain was compared with control strains (Gal4/+ and UAS/+) using one-way ANOVA and Tukey’s test. Genotypes with *p* < 0.05 with both controls are marked as statistically significant change with bold. Degrees of freedom [*F* (DFn, DFd)] are listed for every group.

TABLE S3 Statistical analysis of day-time and night-time sleep. Each experimental strain was compared with control strains (Gal4 and UAS) using one-way ANOVA and Tukey’s test. Genotypes with *p* < 0.05 with both controls are marked as statistically significant change with bold. Degrees of freedom [*F* (DFn, DFd)] are listed for every group.

TABLE S4 Statistical analysis of total activity level. Every experimental strain was compared with control strains (Gal4 and UAS) using one-way ANOVA and Tukey’s test.

TABLE S5 Statistical analysis of PER expression in the clock neurons s-LN_*v*_s and l-LN_*v*_s measured as fluorescence intensity at different time points.
