# Supplementary material for: Communication Among Photoreceptors and the Central Clock Affects Sleep Profile
Source: Front Physiol. 2020 Aug 11;11:993. doi: 10.3389/fphys.2020.00993 (PMC7431659; doi:10.3389/fphys.2020.00993)
Supplement: TABLE S1 — Statistical analysis of the period of locomotor activity rhythm and percent of rhythmic flies. Each experimental strain was compared with control strains (Gal4/+ and UAS/+) using one-way ANOVA and Tukey’s test (for period) or non-parametric Kruskal–Wallis (for % rhythmic). Genotypes with p < 0.05 with both controls are marked as statistically significant changes with bold. Degrees of freedom [F (DFn, DFd)] are listed for every group. [file Table_1.DOCX]

**Table Supplementary 1.**

Statistical analysis of period of locomotor activity and percent of rhythmic flies. Every experimental strain was compared with control strains (Gal4 and UAS) using one way ANOVA and Tukey’s test (for period) or nonparametric Kruskal Wallis (for % rhythmic). Genotypes with p<0.05 with both controls are marked as statistically significant changes with bold. Degrees of freedom [F (DFn, DFd)] are listed for every group.

| **Genotype** | **Period**  **[h]** | **Gal4**  **p-value** | **UAS**  **p-value** | **F**  **(DFn, DFd)** | **% rhythmic** | **Gal4**  **p-value** | **UAS**  **p-value** |
| --- | --- | --- | --- | --- | --- | --- | --- |
| **GMR>*Δcyc******24*** | **22.9** | **<0.0001** | **<0.0001** | 37.11  (2, 212) | **45** | **0.0458** | **0.0286** |
| **GMR>*TeTx*** | 23.8 | 0.7187 | 0.0506 | 2.877  (2, 253) | 95 | 0.106 | 0.8496 |
| ***Rh1*> *Δcyc24*** | 23.7 | 0.2195 | 0.0472 | 2.869  (2, 197) | 77 | 0.0416 | 0.9234 |
| ***Rh1> TeTx*** | 23.5 | 0.5708 | 0.0109 | 5.564  (2, 200) | 100 | >0.9999 | 0.0385 |
| ***Rh3> Δcyc24*** | 23.9 | 0.2675 | 0.015 | 19.99  (2, 230) | 83 | >0.9999 | >0.9999 |
| ***Rh3> TeTx*** | 24.1 | 0.058 | 0.049 | 5.053  (2, 227) | 75 | 0.0387 | 0.3169 |
| ***Rh5> Δcyc24*** | 24.2 | 0.1538 | <0.001 | 13.3  (2, 242) | 80 | 0.6842 | >0.9999 |
| ***Rh5> TeTx*** | 24.3 | 0.059 | 0.037 | 6.786  (2, 269) | 97 | >0.9999 | 0.0637 |
| ***Rh6> Δcyc24*** | 23.6 | 0.0014 | 0.2421 | 6.313  (2, 211) | 99 | >0.9999 | 0.6052 |
| ***Rh6> TeTx*** | 23.6 | 0.0807 | 0.009 | 6.809  (2, 208) | 66 | >0.9999 | >0.9999 |
| ***Rh6>ChatRNAi*** | 23.7 | 0.1363 | <0.001 | 21.04  (2, 193) | 86 | >0.9999 | 0.2775 |
| **L2 *>TeTx*** | 24.0 | 0.8919 | 0.999 | 0.1104  (2, 247) | 96 | >0.9999 | >0.9999 |
