# Supplementary material for: Communication Among Photoreceptors and the Central Clock Affects Sleep Profile
Source: Front Physiol. 2020 Aug 11;11:993. doi: 10.3389/fphys.2020.00993 (PMC7431659; doi:10.3389/fphys.2020.00993)
Supplement: TABLE S2 — Statistical analysis of morning anticipation index (MAI) and evening anticipation index (EAI). Each experimental strain was compared with control strains (Gal4/+ and UAS/+) using one-way ANOVA and Tukey’s test. Genotypes with p < 0.05 with both controls are marked as statistically significant change with bold. Degrees of freedom [F (DFn, DFd)] are listed for every group. [file Table_2.DOCX]

**Table Supplementary 2**

Statistical analysis of Morning Anticipation Index (MAI) and Evening Anticipation Index (EAI). Every experimental strain was compared with control strains (Gal4 and UAS) using one way ANOVA and Tukey’s test. Genotypes with p<0.05 with both controls are marked as statistically significant change with bold. Degrees of freedom [F (DFn, DFd)] are listed for every group.

|  | **MAI** | **GAL4**  **p-value** | **UAS**  **p-value** | **F**  **(DFn, DFd)** | **EAI** | **GAL4**  **p-value** | **UAS**  **p-value** | **F**  **(DFn, DFd)** |
| --- | --- | --- | --- | --- | --- | --- | --- | --- |
| **GMR>*Δcyc24*** | 0.64 | 0.0555 | 0.9679 | 3.995  (2, 187) | **0.9** | **<0.0001** | **<0.0001** | 17.53  (2, 193) |
| **GMR>*TeTx*** | 0.52 | 0.8469 | 0.3277 | 1.432  (2, 267) | **0.9** | **<0.0001** | **<0.0001** | 17.88  (2, 269) |
| ***Rh1*> *Δcyc24*** | 0.7 | <0.0001 | 0.0812 | 9.518  (2, 279) | 0.79 | 0.5339 | 0.4601 | 2.847  (2, 281) |
| ***Rh1> TeTx*** | **0.79** | **<0.0001** | **<0.0001** | 83.34  (2, 382) | 0.8 | 0.8375 | 0.6244 | 1.534  (2, 383) |
| ***Rh3> Δcyc24*** | 0.57 | 0.0632 | 0.4804 | 2.596  (2, 138) | 0.72 | 0.8627 | 0.7196 | 0.723  (2, 147) |
| ***Rh3> TeTx*** | 0.73 | 0.7613 | <0.0001 | 24.25  (2, 239) | 0.7 | 0.6717 | <0.0001 | 12.3  (2, 245) |
| ***Rh5>*** ***Δcyc24*** | 0.59 | 0.5329 | 0.5808 | 0.7263  (2, 202) | 0.78 | 0.3965 | 0.8211 | 1.806  (2, 205) |
| ***Rh5> TeTx*** | 0.56 | 0.0274 | 0.9946 | 3.958  (2, 356) | 0.71 | 0.0552 | 0.2542 | 2.901  (2, 361) |
| ***Rh6> Δcyc24*** | 0.59 | 0.4938 | 0.9785 | 0.7322  (2, 222) | **0.91** | **<0.0001** | **<0.0001** | 29.84  (2, 222) |
| ***Rh6> TeTx*** | 0.6 | 0.0814 | 0.3939 | 10.78  (2, 282) | 0.72 | 0.4844 | 0.9129 | 2.231  (2, 281) |
| ***Rh6>ChatRNAi*** | 0.52 | 0.893 | 0.0012 | 6.835  (2, 174) | **0.88** | **0.017** | **0.0131** | 30,37  (2, 177) |
| **L2 *>TeTx*** | 0.65 | 0.4598 | <0.0001 | 15.79  (2, 358) | **0.87** | **<0.0001** | **<0.0001** | 14.04  (2, 359) |
