# Supplementary material for: Communication Among Photoreceptors and the Central Clock Affects Sleep Profile
Source: Front Physiol. 2020 Aug 11;11:993. doi: 10.3389/fphys.2020.00993 (PMC7431659; doi:10.3389/fphys.2020.00993)
Supplement: TABLE S3 — Statistical analysis of day-time and night-time sleep. Each experimental strain was compared with control strains (Gal4 and UAS) using one-way ANOVA and Tukey’s test. Genotypes with p < 0.05 with both controls are marked as statistically significant change with bold. Degrees of freedom [F (DFn, DFd)] are listed for every group. [file Table_3.DOCX]

|  | **Daytime sleep** | | | **Nighttime sleep** | | |
| --- | --- | --- | --- | --- | --- | --- |
|  | **GAL4**  **p-value** | **UAS**  **p-value** | **F**  **(DFn, DFd)** | **GAL4**  **p-value** | **UAS**  **p-value** | **F**  **(DFn, DFd)** |
| **GMR>*Δcyc24*** | **>0.0001** | **>0.0001** | 39.27  (2, 296) | **>0.0001** | **0.01** | 14.0  (2, 273) |
| **GMR>*TeTx*** | **>0.0001** | **>0.0001** | 67.15  (2, 335) | 0.0271 | 0.052 | 3.908  (2, 335) |
| ***Rh1*> *Δcyc24*** | 0.6152 | 0.873 | 1.656  (2, 316) | **>0.0001** | **>0.0001** | 139.9  (2, 327) |
| ***Rh1> TeTx*** | 0.1098 | 0.9829 | 2.606  (2, 287) | **>0.0001** | **>0.0001** | 107.8  (2,287) |
| ***Rh3> Δcyc24*** | 0.1688 | 0.1109 | 2.046  (2, 285) | **>0.0001** | **>0.0001** | 31.66  (2, 292) |
| ***Rh3> TeTx*** | 0.6001 | 0.8762 | 1.491  (2, 317) | **>0.0001** | **>0.0001** | 135.1  (2, 316) |
| ***Rh5>*** ***Δcyc24*** | **0.0104** | **0.0186** | 5.061  (2, 277) | **0.0001** | **0.0464** | 8.610  (2, 277) |
| ***Rh5> TeTx*** | **0.0282** | **>0.0001** | 9.433  (2,369) | **>0.0001** | **>0.0001** | 66.62  (2, 370) |
| ***Rh6> Δcyc24*** | **0.0065** | **>0.0001** | 12.96  (2, 280) | 0.7534 | 0.9742 | 0.2847  (2, 280) |
| ***Rh6> TeTx*** | **0.00884** | **0.0072** | 6.159  (2, 233) | **0.0216** | **>0.0001** | 29.25  (2, 252) |
| ***Rh6>ChatRNAi*** | **>0.0001** | **>0.0001** | 41.2  (2, 232) | 0.9967 | >0.0001 | 85.9  (2, 232) |
| **L2 *>TeTx*** | **>0.0001** | **>0.0001** | 25.5  (2, 336) | **0.0005** | **>0.0001** | 14.0  (2, 336) |

**Supplementary Table 3**

Statistical analysis of daytime and nighttime sleep. Every experimental strain was compared with control strains (Gal4 and UAS) using one way ANOVA and Tukey’s test. Genotypes with p<0.05 with both controls are marked as statistically significant change with bold. Degrees of freedom [F (DFn, DFd)] are listed for every group.
