# Supplementary material for: Communication Among Photoreceptors and the Central Clock Affects Sleep Profile
Source: Front Physiol. 2020 Aug 11;11:993. doi: 10.3389/fphys.2020.00993 (PMC7431659; doi:10.3389/fphys.2020.00993)
Supplement: TABLE S4 — Statistical analysis of total activity level. Every experimental strain was compared with control strains (Gal4 and UAS) using one-way ANOVA and Tukey’s test. [file Table_4.DOCX]

|  | **GAL4**  **p-value** | **UAS**  **p-value** | **F**  **(DFn, DFd)** |
| --- | --- | --- | --- |
| **GMR>*Δcyc24*** | 0.019 | 0.0044 | 8.037  (2, 297) |
| **GMR>*TeTx*** | 0.039 | 0.0015 | 1.218  (2, 335) |
| ***Rh1*> *Δcyc24*** | <0.0001 | <0.0001 | 50.46  (2, 327) |
| ***Rh1> TeTx*** | <0.0001 | <0.0001 | 30.09  (2, 335) |
| ***Rh3> Δcyc24*** | 0.049 | 0.0043 | 3.481  (2, 295) |
| ***Rh3> TeTx*** | 0.0088 | <0.0001 | 24.58  (2, 314) |
| ***Rh5>*** ***Δcyc24*** | 0.0004 | <0.0001 | 12.01  (2, 318) |
| ***Rh5> TeTx*** | 0.0004 | <0.0001 | 33.14  (2, 372) |
| ***Rh6> Δcyc24*** | >0.9999 | 0.026 | 1.726  (2, 266) |
| ***Rh6> TeTx*** | 0.5985 | 0.0117 | 8.325  (2, 254) |
| ***Rh6>ChatRNAi*** | 0.0001 | 0.0002 | 34.27  (2, 237) |
| **L2 *>TeTx*** | 0.0005 | 0.0074 | 21.72  (2, 336) |

**Supplementary Table 4**

Statistical analysis of total activity level. Every experimental strain was compared with control strains (Gal4 and UAS) using one way ANOVA and Tukey’s test.
