# Supplementary material for: Communication Among Photoreceptors and the Central Clock Affects Sleep Profile
Source: Front Physiol. 2020 Aug 11;11:993. doi: 10.3389/fphys.2020.00993 (PMC7431659; doi:10.3389/fphys.2020.00993)
Supplement: TABLE S5 — Statistical analysis of PER expression in the clock neurons s-LNvs and l-LNvs measured as fluorescence intensity at different time points. [file Table_5.DOCX]

|  | **s-LNv** | | **l-LNv** | |
| --- | --- | --- | --- | --- |
|  | **Gal4/+**  **p-value** | **UAS/+**  **p-value** | **Gal4/+**  **p-value** | **UAS/+**  **p-value** |
| **GMR>TeTx** | | | | |
| **ZT0** | **<0.0001** | **<0.0001** | **<0.0001** | **<0.0001** |
| **ZT4** | 0.2452 | 0.7037 | **<0.0001** | **0.0117** |
| **ZT8** | 0.0623 | <0.0001 | 0.0011 | 0.2732 |
| **ZT12** | 0.4126 | 0.976 | 0.9396 | 0.9395 |
| **ZT16** | **<0.0001** | **<0.0001** | **0.014** | **0.007** |
| **ZT20** | **<0.0001** | **<0.0001** | **<0.0001** | **<0.0001** |
| **GMR>cycΔ24** | | | | |
| **ZT0** | **<0.0001** | **<0.0001** | **<0.0001** | **<0.0001** |
| **ZT4** | **0.0279** | **<0.0001** | <0.0001 | 0.0263 |
| **ZT8** | **0.0125** | **0.0125** | 0.6611 | 0.0049 |
| **ZT12** | 0.6778 | 0.6778 | 0.9498 | 0.1309 |
| **ZT16** | **<0.0001** | **<0.0001** | **0.0053** | **<0.0001** |
| **ZT20** | **<0.0001** | **<0.0001** | **<0.0001** | **<0.0001** |

Supplementary Table 5

Statistical analysis of PER expression in clock neurons s-LN_v_s and l-LN_v_s.
